# Probing the 3D molecular and mineralogical heterogeneity in oil reservoir rocks at the pore scale

**DOI:** 10.1038/s41598-019-44763-6

**Published:** 2019-06-04

**Authors:** Guilherme José Ramos Oliveira, Paula Campos de Oliveira, Rodrigo Surmas, Leandro de Paulo Ferreira, Henning Markötter, Nikolay Kardjilov, Ingo Manke, Luciano Andrey Montoro, Augusta Isaac

**Affiliations:** 1 Universidade Federal de Minas Gerais, Department of Metallurgical and Materials Engineering, Belo Horizonte, 31270-901 Brazil; 2 Petróleo Brasileiro S.A. - PETROBRAS, Research and Development Center, Cidade Universitária, Rio de Janeiro, 21941-970 Brazil; 3 Universidade Federal de Minas Gerais, Department of Chemistry, Belo Horizonte, 31270-901 Brazil; 4 Institute of Applied Materials, Helmholtz-Zentrum Berlin, Hahn-Meitner-Platz 1, 14109 Berlin, Germany

**Keywords:** Fossil fuels, Energy science and technology

## Abstract

Innovative solutions have been designed to meet the global demand for energy and environmental sustainability, such as enhanced hydrocarbon recovery and geo-sequestration of CO_2_. These processes involve the movement of immiscible fluids through permeable rocks, which is affected by the interfacial properties of rocks at the pore scale. Overcoming major challenges in these processes relies on a deeper understanding about the fundamental factors that control the rock wettability. In particular, the efficiency of oil recovery strategies depends largely on the 3D wetting pattern of reservoir rocks, which is in turn affected by the adsorption and deposition of ‘contaminant’ molecules on the pores’ surface. Here, we combined high-resolution neutron tomography (NT) and synchrotron X-ray tomography (XRT) to probe the previously unobserved 3D distribution of molecular and mineralogical heterogeneity of oil reservoir rocks at the pore scale. Retrieving the distribution of neutron attenuation coefficients by Monte Carlo simulations, 3D molecular chemical mappings with micrometer dimensions could be provided. This approach allows us to identify co-localization of mineral phases with chemically distinct hydrogen-containing molecules, providing a solid foundation for the understanding of the interfacial phenomena involved in multiphase fluid flow in permeable media.

## Introduction

Technological solutions have been designed to meet the global demand for energy and environmental sustainability, including enhanced hydrocarbon recovery^1–4^, geo-sequestration of carbon dioxide^5,6^_,_ security of CO_2_ storage^7,8^, remediation of nonaqueous phase liquid contaminants in aquifers^9^. Overcoming major challenges in these processes relies, to a significant extent, on the understanding of how immiscible fluids interact with solid surface within porous rocks and soil^10–12^.

Interfacial phenomena in multiphase fluid flow become quite complex if the porous rock is chemically and physically heterogeneous. A further complication is the alteration of the original wettability of pore surfaces caused by the formation of “contaminant” layers throughout the rock^11,13^. For instance, the adsorption or deposition of heavy components of crude oil on a mineral surface has long been recognized as wettability modifiers^14–20^.

Significant progress towards a deeper understanding of wetting behavior of porous rocks was made with the employment of three-dimensional (3D) X-ray imaging techniques^21–27^. X-ray microtomography (XRT), in particular, allowed for measuring the *in situ* wettability of reservoir rocks at subsurface conditions^13,21,25,27^. With the use of XRT approaches, contact angle measurements between fluids and pore surfaces provided direct evidence of the mixed-wettability even in mineralogically homogeneous rocks^11,13^. Recently, the mixed-wet state of porous rocks has been associated with variations in surface roughness^11,13^. It was found that the contact angle tends to be lower on rougher surfaces due to the accumulation of water in crevices, what makes the surface more hydrophilic^11^.

Despite the great advances achieved with XRT approaches, imaging of molecular substances (such as water and oil) using X-ray absorption-based techniques provide poor contrast^28^. These techniques are based on the differences in attenuating power of the analyzed materials^29^, resulting on imaging contrast during acquisition. As the x-ray beam passes through the sample, photons get absorbed; this is known as attenuation. Much of this effect is related to the photoelectric effect. The probability of photoelectric absorption is strongly related with atomic number (Z) and is dependent on density (ρ) and thickness of the materials present in the sample^29^. In the context of porous rocks, XRT allows for imaging the 3D distribution of minerals, pores, and fractures usually with excellent contrast^30^; however, imaging molecular substances that exhibit low values of Z and ρ results in a poor contrast^28,29^.

Unlike X-rays, neutrons interact weakly with most minerals, but strongly with small amounts of hydrogen or, more precisely, hydrogen-containing substances^31^. However, neutron imaging has been limited to the investigation of coarse-grained materials due to its low spatial resolution^32^. Current developments of more sensitive detectors, such as cooled CCD detectors with an improved sensitivity, has led to significant improvements in spatial resolution reaching about 10 µm^33^. High-resolution neutron tomography (NT), with a level now comparable with XRT, emerges as a powerful technique to study the 3D distribution of hydrogen-containing phases (such as water and oil) in real systems *in situ*. This capability results from the high interaction cross section of hydrogen atoms for cold neutron scattering^31^.

Accordingly, we present a novel approach to probe the 3D interfacial characteristics of complex porous rocks using combined high-resolution neutron (NT) and synchrotron X-ray tomography techniques. The achieved spatial resolution of the neutron tomography (pixel size of 10 µm) combined to its extremely high sensitivity to hydrogen allowed us to resolve adsorbed or deposited molecular “contaminant” layers on the pore surface. Retrieving the 3D distribution of neutron attenuation coefficients of reservoir rocks by Monte Carlo simulations, we could unambiguously distinguish between water and oil phase through the sample volume. The coupling of high-resolution XRT and NT can be used as a platform to explore a huge range of interfacial phenomena, providing information that is not possible to obtain using other characterization techniques.

## Results and Discussion

### Conventional characterization of a complex porous rock

**Figure 1:**
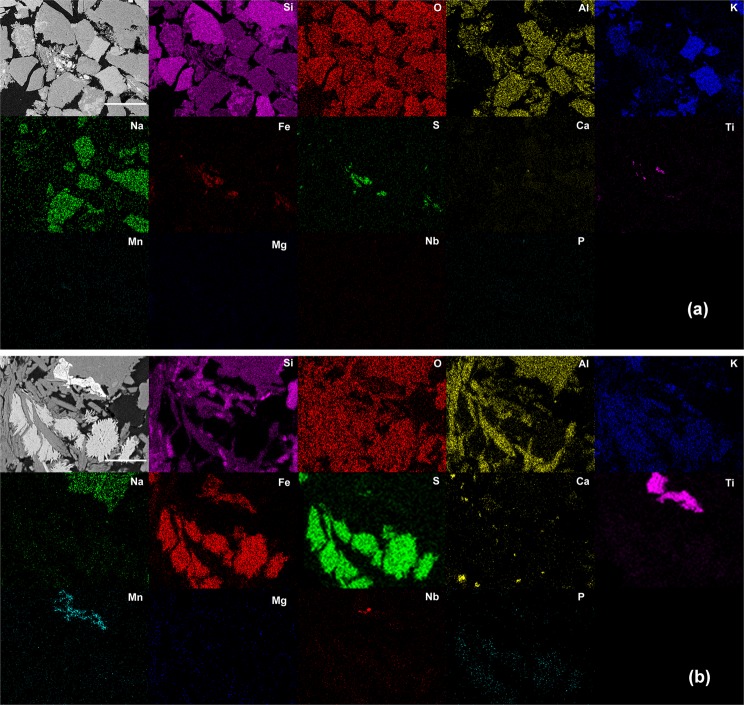
SEM image (from backscattered electron detection) of the sandstone rock and associated elemental maps. (**a**) Large grains mainly composed of Si, O, Al, K, and Na. Scale bar: 300 µm. (**b**) Enlarged view of the central region in (**a**). Scale bar: 50 µm.

**Figure 2:**
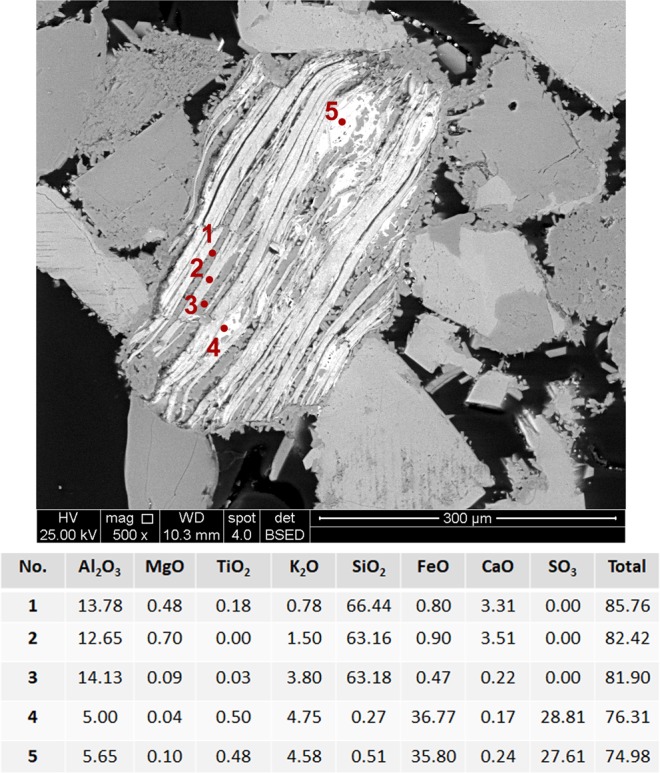
SEM image of a mica grain with inclusions acquired from backscattered electron detection with punctual regions analyzed by electron probe microanalysis (WDS – wavelength dispersive spectroscopy). The inserted table shows the quantitative analysis of elemental content represented, as usual, as simple oxide phases. Numbers in the figure indicate the analyzed points.

We investigated a fine-grained sandstone reservoir rock, which contains predominantly quartz (SiO_2_) and orthoclase (KAlSi_3_O_8_), with minor amounts of muscovite (KAl_2_(Si_3_Al)O_10_(OH,F)_2_) and albite (NaAlSi_3_O_8_). Elemental composition maps obtained by scanning electron microscopy coupled to energy dispersive x-ray spectroscopy (SEM-EDS) confirms that larger grains of about 100–250 µm were mostly composed of Si, O, Al, and K (Fig. 1a), whereas localized concentrations of Fe associated with S as well as Ti, Mn, Nb, and Ca could be noticed for smaller structures (Fig. 1b). Punctual analysis using electron probe microanalysis (EPMA) were performed in those small structures (Fig. 2). Figure 2 shows a representative mica grain holding inclusions in the interlayer region. These inclusions are mostly composed of species with high concentration of Fe and S (indicated by numbers 4 and 5 in Fig. 2).

**Figure 3:**
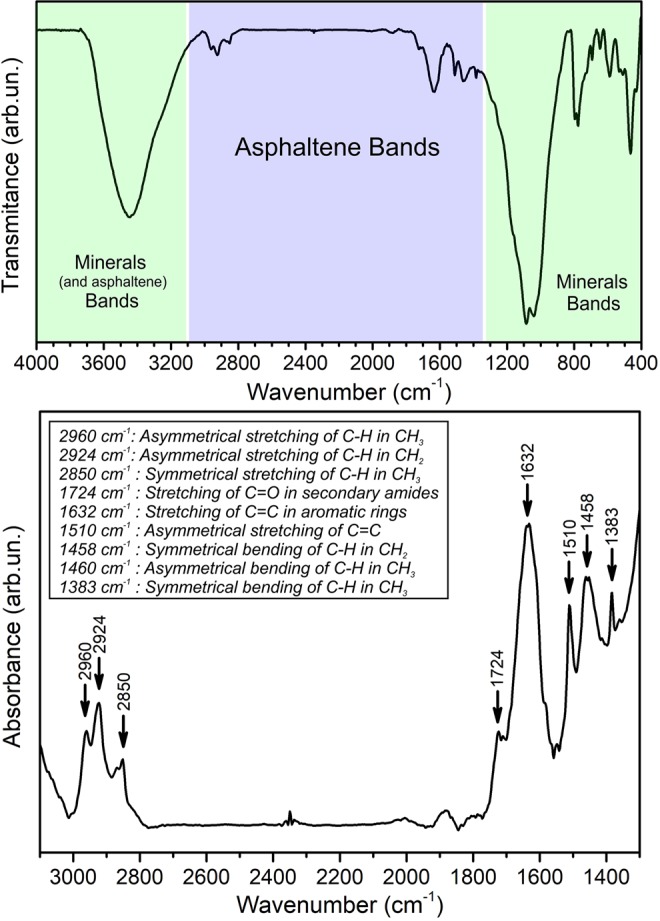
Infrared spectra (FTIR) acquired from the pulverized porous rock, evidencing minerals phases and asphaltene (upper spectra). The asphaltene bands are assigned on the bottom spectra.

In addition, infrared spectroscopy was applied to probe que chemical components in a pulverized rock sample. The analyzed specimen was prepared as a KBr pellet and the collected spectra is presented in Fig. 3. The whole view from 400 to 4000 cm^−1^ evidence different bands associated with both the minerals phases and asphaltenes. The strong bands from 400–1300 cm^−1^ region are associated with the minerals, quartz, orthoclase and albite^19,34,35^. The wide band around 3500 cm^−1^ is attributed to the typical –OH stretching, present in the muscovite phase, water and asphaltenes. On the central region from 1300–3000 cm^−1^ are observed several medium and week bands attributed to the minor component asphaltenes. The asphaltenes are a complex mixture of organic compounds found in the crude oil which are often impregnated into these porous rocks. The organic structure is constituted by aliphatic and romantic chains with oxygen, nitrogen, and sulphur. These asphaltene bands are assigned on the table of Fig. 3, resulting mainly from stretching and bending of such aromatic and aliphatic structures^19,34,35^.

### Revealing the rock heterogeneity by combined neutron and X-ray tomography

**Figure 4:**
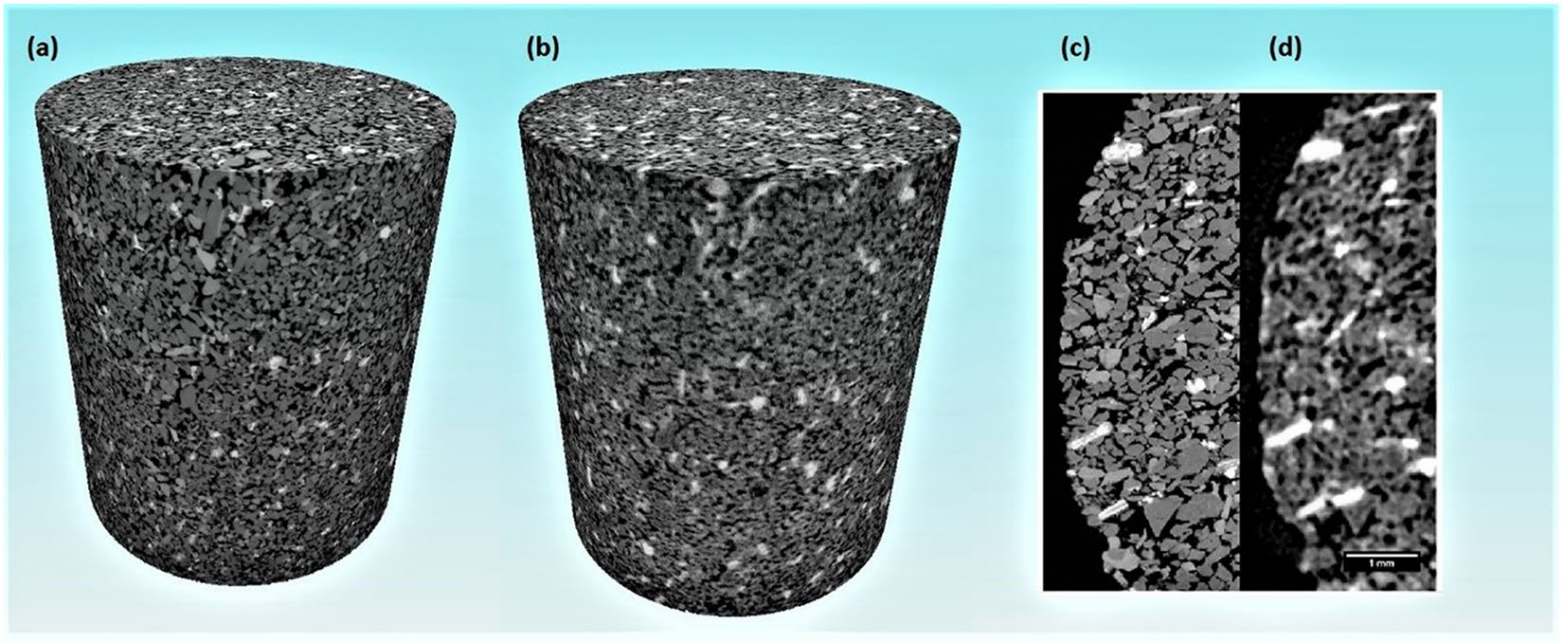
Three-dimensional images of the sandstone rock obtained using synchrotron X-ray tomography (**a**) and neutron tomography (**b**). Comparison between cross-sectional images of the synchrotron X-ray tomography volume (**c**) and neutron tomography data (**d**). The upper diameter and height of the sample are about 6 and 14 mm, respectively.

The sandstone rock sample was evaluated by both XRT and NT techniques, providing complementary information on its main constituents (Fig. 4). The image contrast obtained using X-rays reveals variations in density and atomic number; and the contrast provided by neutrons reveals the distribution of hydrogen-containing materials such as water and oil^33^.

From the intensity profile obtained by synchrotron XRT, at least three phases and the pore space could be clearly distinguished (Figs 4 and S2). Based on the theoretical attenuation coefficient (µ_th_) of minerals^36^, we could associate the brightest regions of the images mostly to mica and inclusions (Fig. S2-d). Dark gray covers quartz and albite together (Fig. S2-b); these minerals could not be distinguished due to their small difference in attenuation coefficient^36^. Intermediate gray levels correspond to orthoclase (Fig. S2-c)^36^. The quantitative analysis of the X-ray images showed that the volume fractions of quartz together with albite, orthoclase, and mica are 51.2%, 21.7%, and 2.2%, respectively. The total porosity computed from the X-ray images is about 24.9% (Table 1). By considering only the mineral phases (excluding pores), the volume fractions of quartz plus albite, orthoclase, and mica are 68.2%, 28.9%, and 2.9%. These results are in good agreement with the Rietveld X-ray diffraction quantitative analysis, which indicates 70.2% of quartz plus albite and 30.3% of orthoclase. These results are exhibited in Table S1 (Supplementary Information Appendix).

**Table 1:** Quantitative analysis of the X-ray images revealing the volume fraction of the mineral phases and pores.

Segmented phases	Volume fraction (%)	
Minerals	**Quartz**	51.2 ± 1.5
	**Albite**	
	**Orthoclase**	21.7 ± 1.5
	**Muscovite**	2.2 ± 1.5
Pores		25.0 ± 1.5

Complementing the X-ray results, neutron imaging reveals the distribution of hydrogen-rich materials in the sample (bright regions of Fig. 4b). For providing chemical information on the constituents of the sandstone sample, we computed the neutron attenuation coefficient (Σ_m_) for each voxel (11 µm × 11 µm × 11 µm) of the reconstructed volume using Monte Carlo simulations^37^. This approach enables us to unambiguously distinguish the hydrogen-containing substances between hydrous minerals (such as mica), aqueous and non-aqueous phases because their attenuation properties differ largely in a cold monochromatic neutron beam^38^.

**Figure 5:**
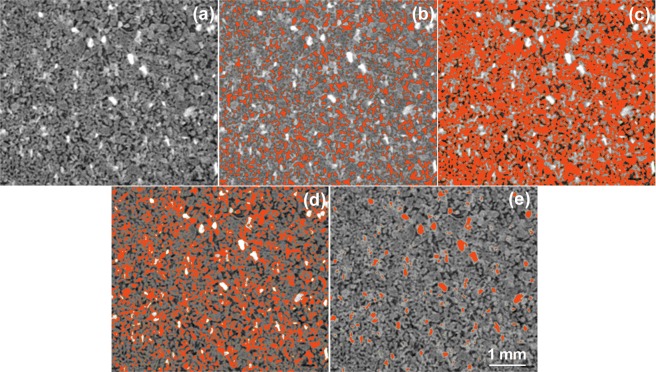
Neutron image segmentation of the sandstone rock sample. The distribution of measured attenuation coefficients of a selected representative image (**a**) is divided in four classes: (**b**) Σ_m_ < 0.12 cm^−1^, (**c**) 0.12 ≤ Σ_m_ < 0.40 cm^−1^, (**d**) 0.40 ≤ Σ_m_ < 0.65 cm^−1^, and (**e**) Σ_m_ ≥ 0.65 cm^−1^.

The distribution of the measured attenuation coefficients varies from values typical of pores (the absence of materials, Σ_m_ = 0 cm^−1^) to those produced by highly attenuating materials to neutrons (Σ_m_ ≈ 1.70 cm^−1^) (Fig. 5). Four classes of Σ_m_ values could be segmented: (i) Σ_m_ < 0.12 cm^−1^ accounting for 16.5% of the sample volume (highlighted in Fig. 5b); (ii) 0.12 ≤ Σ_m_ < 0.40 cm^−1^ with a volume fraction of 55.3% (Fig. 5c); (iii) 0.40 ≤ Σ_m_ < 0.65 cm^−1^ with a volume fraction of 24.2% (Fig. 5d), and (iv) Σ_m_ ≥ 0.65 cm^−1^ exhibiting nearly 4% of the total volume (Fig. 5e).

It should be noticed that the predominant minerals of the rock (i.e., quartz, orthoclase, and albite) exhibits values of Σ_m_ between 0.12 cm^−1^ and 0.65 cm^−1^ (Fig. 5c). The average Σ_m_ of these limits (0.265 cm^−1^) agrees well with the theoretical attenuation coefficients (Σ_th_) of quartz (Σ_th_ = 0.287 cm^−1^), orthoclase (Σ_th_ = 0.260 cm^−1^), and albite (Σ_th_ = 0.280 cm^−1^)^39^. Voxels exhibiting Σ_m_ in the range of 0.40 and 0.65 cm^−1^ can be explained by the presence of hydrogenous substances in small pores which cannot be resolved by neutron tomography due to its limited spatial resolution (Fig. 5d). The attenuation coefficients ranging from 0.65 cm^−1^ to 1.7 cm^−1^ correspond to hydrogen-rich materials with varying concentration of hydrogen (Fig. 5e). This range of Σ_m_ includes hydroxide minerals such as muscovite (Σ_th_ is 0.935 cm^−1^) and aggregations of crude oil components. From the FTIR analysis (Fig. 3), we could ascribe these aggregations to asphaltenes^40^. In addition, the fact that asphaltene is not a single compound, but a compound class, may explain the observed variations in Σ_m_ in the range of 0.65 cm^−1^ to 1.7 cm^−1^ (Fig. 5d).

Asphaltenes comprises a complex mixture formed by molecules which contrast sharply in polarity and structure from the majority of the crude oil components, which accounts for their predisposition to self-aggregation and adsorption at surfaces^19,40^. In practice, this leads to undesirable consequences, such as the deposition and wettability alteration during crude oil extraction, production, and refining^19,41^. Several authors investigated the tendency for wettability alteration with the adsorption/deposition of asphaltenes on flat and homogeneous mineral surfaces by contact angle measurements and atomic force microscopy^15,19,40–44^. Unlike the previous studies, this work presents a novel approach to directly visualize the 3D distribution of crude oil components and aqueous phases. This approach will enable to study the influence adsorbed/deposited water or oil components on fluids flow and finally the displacement efficiency.

**Figure 6:**
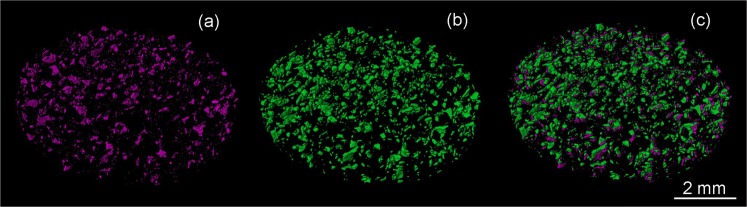
Direct evidence of the interaction between mica grains with crude oil components. Three-dimensional images of a slab of the sandstone rock. (**a**) 3D X-ray surface-rendered image indicating the distribution of the mica grains. (**b**) 3D neutron surface-rendered image highlighting the distribution of aggregations of oil components. (**c**) Both images superimposed, where the good matching suggests a mica-oil interaction.

**Figure 7:**
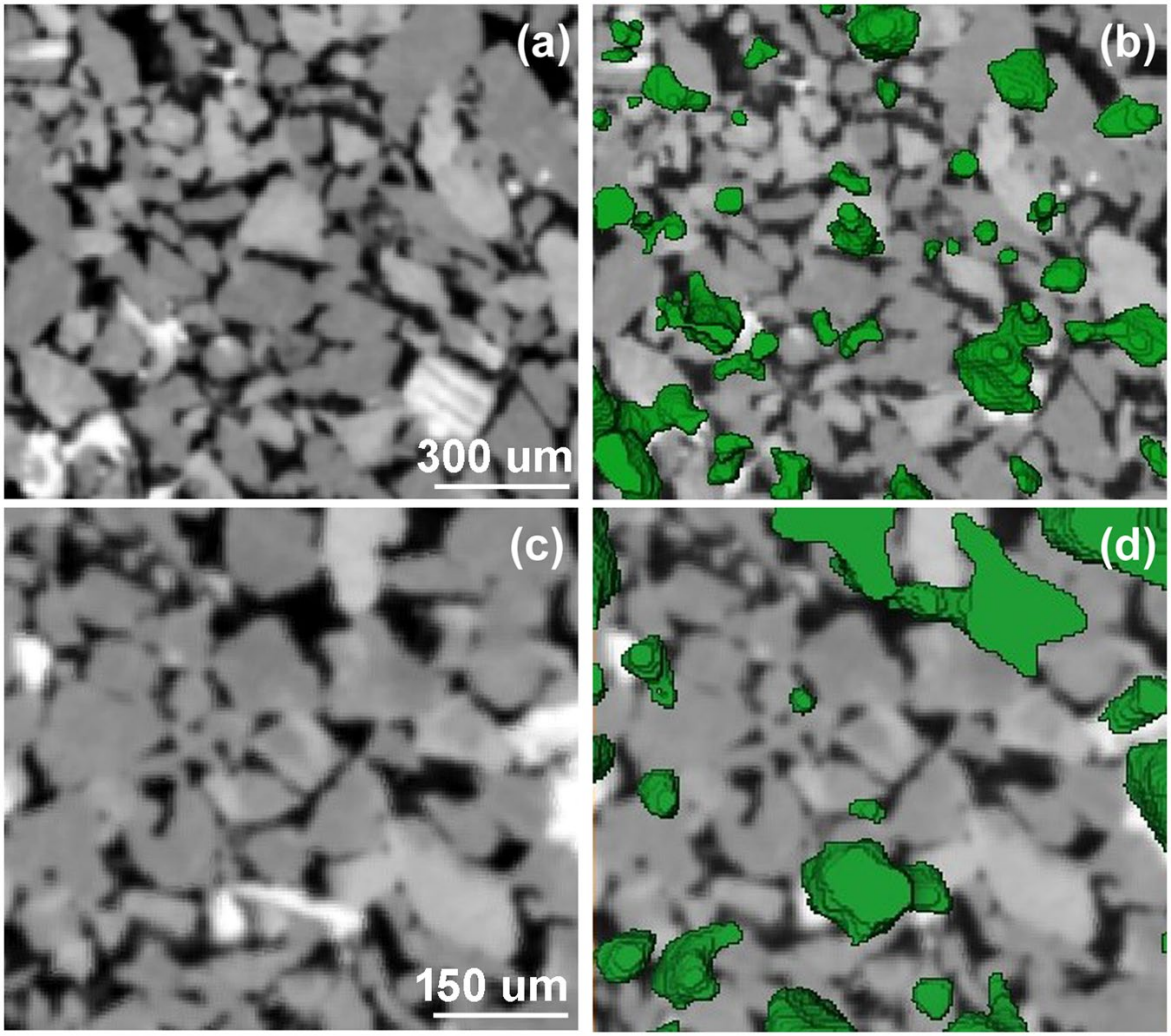
Pore filling and mica-asphaltenes interactions. X-ray cross-sectional image (**a**) and this image overlaid by the rendered surface of oil aggregations extracted from the neutron volume (**b**). Higher magnification of the figures (**a**,**b**) are displayed in (**c**,**d**), respectively.

In order to reveal the location of adsorbed and/or deposited asphaltenes in the sandstone reservoir rock, 3D rendered X-ray and neutron volumes were superposed as shown in Fig. 6. Figure 6a exhibits the 3D rendered surface of the high-absorbing particles to X-rays (mica grains and heavy inclusions); Fig. 6b, the aggregations of asphaltenes extracted from the neutron volume; and Fig. 6c, the superposed images revealing that asphaltenes are mostly located on the mica grains and their surroundings. The rendered surface of oil aggregations was also superimposed on the X-ray cross-section in Fig. 7. Besides the interaction between the mica grains and asphaltenes, we could observe that some pores were filled with oil (Fig. 7). This agrees well with the reduced porosity computed for the neutron volume compared to the X-ray data (16.5% and 25.0%, respectively). The mica-asphaltenes interaction can be rationalized in terms of the mica structure and its chemical properties. Mica is a phyllosilicate mineral of aluminum and potassium with a highly perfect basal cleavage and a lamellar morphology. It is well known that this lamellar structure promotes strong interfacial binding due to van der Waals (vdW) interactions, for example, with graphene layers^15^. These vdW interactions can be the predominant adhesion forces between the mica grains and the asphaltenes.

In summary, we could distinguish between two types of inclusions containing hydrogen: i. fine inclusions within micropores (with size below the resolution of the neutron imaging) in minerals with a volume fraction of about 24.2% (Table 2); and ii. large ones with approximately 2% volume fraction (subtract the volume fraction of hydroxide minerals from the voxels exhibiting 0.65 < Σ_m_ ≤ 1.7 cm^−1^). Due to the presence of adsorbed and/or deposited crude oil components, we can infer that a significant portion of the minerals surface in the rock sample is preferentially wetting to a nonaqueous phase. This information is of paramount importance for the modelling and quantification of flow and transport in porous rocks. Therefore, the combined X-ray and neutron imaging approach provides a deeper understanding of the rock heterogeneity and the factors that affect its wettability pattern. The ability to properly characterize such complex systems at the pore level can be used to explore a huge range of phenomena in the energy and environmental arenas.

**Table 2:** Quantitative analysis of the neutron imaging revealing the volume fraction of the segmented phases.

Segmented phases	Volume fraction (%)
Quartz, albite, and orthoclase	55.3 ± 2.0
Minerals with fine hydrogen-containing inclusions	24.2 ± 2.0
Large hydrogen-rich inclusions and hydroxide minerals	4 ± 1.0
Pores	16.5 ± 2.0

## Conclusions

In this work, we present a novel approach for probing *in situ* the physical-chemical characteristics of complex porous rocks by the combined use of high-resolution X-ray and neutron tomography. Further, the distribution of measured neutron attenuation coefficients of the sample was retrieved from the intensity profiles using Monte Carlo simulations. This permitted to chemically distinguish the hydrogen domains between water, crude oil components, and hydroxide minerals (such as muscovite).

Here, we characterized a fine-grained and mineralogically heterogeneous sandstone rock from a producing oil reservoir. This sample is composed mostly of quartz, orthoclase, albite, and narrow flakes of mica. The complementarity of X-ray and neutron imaging enabled us to reveal for the first time the 3D distribution of crude oil components (asphaltenes) and their strong preference for the mica grains. Additionally, 10% of the porosity observed in the X-ray images were filled with crude oil components.

Besides the wetting behavior of natural porous systems, this approach paves the way for understanding rocks and fluids interactions. Such situations are conceivable across a broad range of extraction, separation, or contamination processes or phenomena involving immiscible fluids.

## Materials and Methods

### Porous rocks

Three sandstone core samples of about 7 mm in diameter and 10 mm in height from a producing oil reservoir were chosen for this study. They are principally composed of quartz (SiO_2_) and orthoclase (KAlSi_3_O_8_), albite (NaAlSi_3_O_8_), and minor amounts of muscovite (KAl_2_(Si_3_Al)O_10_(OH,F)_2_). The diffraction pattern of this sample is presented in Fig. S1 in the SI Appendix. In addition, quantitative phase analysis of the sample was performed using Rietveld refinement. Based on the diffraction pattern of the sandstone rock, the volume fraction of quartz, albite, and orthoclase were 39.9%, 30.3%, and 30.4%. respectively. The low amount of muscovite in the sample could not be accurately quantified by XRD.

### Chemical and structural characterization of the sandstone rocks

Elemental mapping analysis of the rock samples were obtained using energy-dispersive X-ray spectroscopy (EDS) and wavelength-dispersive X-ray spectroscopy (WDS) in a JEOL 8900 electron microprobe. The infrared absorption spectra were recorded with a spectrometer 1760X Perkin-Elmer with Fourier Transform in the region of 400–4000 cm^−1^ with a resolution of 0.01 cm^−1^ by using KBr pellets.

### Imaging rock and fluids interactions

We combined high-resolution neutron and synchrotron X-ray tomography to explore rock-fluid interactions in an oil reservoir rock. Whereas neutron imaging provides high contrast of hydrogen-rich substances^45^, the contrast of the images obtained using X-rays allows to differentiate mineral phases based on variations in both atomic number and density^46^.

Tomography experiments were performed at Helmholtz-Zentrum-Berlin für Materialien und Energie (HZB), in Berlin/Germany^33,47^. HZB operates two scientific large-scale facilities for materials investigation: the research reactor BER II for experiments with neutrons and the third-generation synchrotron photon source BESSY II. Fully dedicated instruments for materials imaging are provided by these facilities; particularly, the BAMline/BESSY II^47^ and the CONRAD-2/BER II^33^. The neutron and X-ray images achieved a spatial resolution of approximately 22 μm (11.2 μm/pixel) and 4 μm (2.2 μm/pixel), respectively. Further details on the experimental conditions are presented in Table 3.

**Table 3:** Experimental conditions used in the tomography experiments.

Tomography	Neutrons	Synchrotron X-ray
Instrument	CONRAD-2/BERII/HZB^33^	BAMline/BESSY-II/HZB^47^
Pixel size [µm]	11.2	2.2
Exposure time [s]	15/projection	2.5/projection
No. of projections	1800	3600
Energy/Wavelength	2–6 Å	21 keV
Detector resolution	2048 × 2048	4008 × 2672
Scintillator/detector	20 µm Gadox	60 µm CdWO_4_

The visualization and quantitative analysis of the reconstructed volumes were performed using Thermo Scientific™ Avizo™ Software (ThermoFisher Scientific, Oregon/USA). For the simultaneous visualization of the mineralogical and molecular characteristics of the rock sample, the X-ray and neutron volumes were aligned into the same coordinate system. For that, it was used a reliable image registration method available in Thermo Scientific™ Avizo™ Software. The registration of the two 3D data sets (i.e., XRT and NT volumes) was based on the position of specific pores which were used as markers. Independently on the imaging technique, pores always correspond to the darkest voxels. Thus, a set of large pores were selected for the image registration procedure and the centers of gravity (CG) of individual pores were computed for the XRT and NT volumes. The XRT volume was then rotated and translated with respect to the NT volume (reference) until a good matching is obtained based on the position of CGs of the individual pores. The optimized alignment was determined by a volume-matching quality measure which is a function of the differences in CG position of each pore.

For measuring the uncertainties of the quantitative analyses based on the XRT and NT data sets, voxel intensities (gray values) of different grains associated with a specific phase were used to calculate the average value and standard deviation. Approximately, 2500 voxel intensities were analyzed for each phase. Subsequently, volume fractions and porosity were recalculated varying the user-defined threshold limits by subtracting and adding the standard deviation. This process led to the uncertainties expressed in Tables 1 and 2.

The chemical analysis of the neutron tomography data was supported by Monte Carlo simulations which provided the theoretical values for the neutron attenuation coefficients of the minerals presented in the sample. The complex mineral composition and the multiparameter experimental conditions required more sophisticated model of the neutron interaction with matter. For this purpose, it was used the Monte Carlo N-Particle (MCNP) Transport Code (Los Alamos National Laboratory, U.S. Department of Energy’s NNSA) with full description of the material (composition and density), the geometrical parameters (sample size, source-sample and sample-detector distances) and the beam properties (neutron spectrum and beam divergence)^37^. The value of the attenuation coefficient for each mineral was calculated in cm^−1^ units; therefore, the voxel values from the tomography experiments were converted in the same units (cm^−1^). A computation procedure based on thresholding of the experimental tomography volumes using the theoretical values from the MCNPX simulations allowed for retrieving the 3D distribution of the different minerals and molecular substances in the rock sample. In addition, cross-check calculations were performed using the Neutron activation and scattering calculator provided as online service by NIST^39^; the theoretical attenuation coefficients were computed using a single neutron wavelength (0.25 nm – the maximum in the neutron spectrum) without any geometrical parameters (Table S2, SI Appendix). The results from the two methods agreed in the range of 2%, confirming the reliability of the calculated values for the mineral’s attenuation coefficients.

## Supplementary information

Supplementary Information

## References

[1] Lai J (2018). A review on pore structure characterization in tight sandstones. Earth-Science Reviews.

[2] De Almeida, J. M. & Miranda, C. R. Improved oil recovery in nanopores: NanoIOR. *Sci*. *Rep*. **6** (2016). 10.1038/srep28128 PMC4913300 27319357

[3] Desmond, J. L. *et al*. Organic-Silica Interactions in Saline: Elucidating the Structural Influence of Calcium in Low-Salinity Enhanced Oil Recovery. *Sci*. *Rep*. **7** (2017). 10.1038/s41598-017-10327-9 PMC5591284 28887490

[4] Lee, T., Bocquet, L. & Coasne, B. Activated desorption at heterogeneous interfaces and long-time kinetics of hydrocarbon recovery from nanoporous media. *Nat*. *Commun*. **7** (2016). 10.1038/ncomms11890 PMC4919511 27327254

[5] Abidoye LK, Khudaida KJ, Das DB (2015). Geological carbon sequestration in the context of two-phase flow in porous media: A review. Critical Reviews in Environmental Science and Technology.

[6] Lal R (2008). Sequestration of atmospheric CO2 in global carbon pools. Energy Environ. Sci..

[7] Jia B, Tsau J-S, Barati R (2019). A review of the current progress of CO2 injection EOR and carbon storage in shale oil reservoirs. Fuel.

[8] Leung DYC, Caramanna G, Maroto-Valer MM (2014). An overview of current status of carbon dioxide capture and storage technologies. Renew. Sustain. Energy Rev..

[9] Adadevoh JST, Ramsburg CA, Ford RM (2018). Chemotaxis Increases the Retention of Bacteria in Porous Media with Residual NAPL Entrapment. Environ. Sci. Technol..

[10] Holtzman, R. Effects of Pore-Scale Disorder on Fluid Displacement in Partially-Wettable Porous Media. *Sci. Rep*. **6** (2016). 10.1038/srep36221 PMC5080577 27782194

[11] AlRatrout, A., Blunt, M. J. & Bijeljic, B. Wettability in complex porous materials, the mixed-wet state, and its relationship to surface roughness. *Proc*. *Natl*. *Acad*. *Sci*. 201803734, 10.1073/pnas.1803734115 (2018). 10.1073/pnas.1803734115 PMC6130345 30120127

[12] Blunt, M. J. *Multiphase Flow in Permeable Media*, 10.1017/9781316145098 (Cambridge University Press, 2017).

[13] Alhammadi, A. M., Alratrout, A., Singh, K., Bijeljic, B. & Blunt, M. J. *In situ* characterization of mixed-wettability in a reservoir rock at subsurface conditions. *Sci*. *Rep*. **7** (2017). 10.1038/s41598-017-10992-w PMC5589931 28883407

[14] Syunyaev RZ, Balabin RM, Akhatov IS, Safieva JO (2009). Adsorption of petroleum asphaltenes onto reservoir rock sands studied by near-infrared (NIR) spectroscopy. In Energy and Fuels.

[15] Adams JJ (2014). Asphaltene adsorption, a literature review. Energy and Fuels.

[16] Hematfar Vahid, Maini Brij, Chen Zhangxing(John) (2018). Experimental investigation of asphaltene adsorption in porous media due to solvent injection and effects on relative permeability. International Journal of Multiphase Flow.

[17] Natarajan A (2014). Understanding mechanisms of asphaltene adsorption from organic solvent on mica. Langmuir.

[18] Jada A, Debih H (2009). Hydrophobation of clay particles by asphaltenes adsorption. Compos. Interfaces.

[19] Gonzalez V, Taylor SE (2016). Asphaltene adsorption on quartz sand in the presence of pre-adsorbed water. J. Colloid Interface Sci..

[20] Xie X, Morrow NR, Buckley JS (2002). Contact angle hysteresis and the stability of wetting changes induced by adsorption from crude oil. J. Pet. Sci. Eng..

[21] Berg S (2013). Real-time 3D imaging of Haines jumps in porous media flow. Proc. Natl. Acad. Sci. USA.

[22] Wildenschild D, Sheppard AP (2013). X-ray imaging and analysis techniques for quantifying pore-scale structure and processes in subsurface porous medium systems. Adv. Water Resour..

[23] Singh K, Bijeljic B, Blunt MJ (2016). Imaging of oil layers, curvature and contact angle in a mixed-wet and a water-wet carbonate rock. Water Resour. Res..

[24] Andrew, M., Menke, H., Blunt, M. J. & Bijeljic, B. The Imaging of Dynamic Multiphase Fluid Flow Using Synchrotron-Based X-ray Microtomography at Reservoir Conditions. *Transp. Porous Media* **110** (2015).

[25] Rücker M (2015). From connected pathway flow to ganglion dynamics. Geophys. Res. Lett..

[26] Menke HP, Andrew MG, Blunt MJ, Bijeljic B (2016). Reservoir condition imaging of reactive transport in heterogeneous carbonates using fast synchrotron tomography - Effect of initial pore structure and flow conditions. Chem. Geol..

[27] Pak T, Butler IB, Geiger S, van Dijke MIJ, Sorbie KS (2015). Droplet fragmentation: 3D imaging of a previously unidentified pore-scale process during multiphase flow in porous media. Proc. Natl. Acad. Sci..

[28] Momose A (2005). Recent advances in X-ray phase imaging. Japanese Journal of Applied Physics, Part 1: Regular Papers and Short Notes and Review Papers.

[29] Maire E, Withers PJ (2014). Quantitative X-ray tomography. Int. Mater. Rev..

[30] Lai, J. *et al*. Three-dimensional quantitative fracture analysis of tight gas sandstones using industrial computed tomography. *Sci*. *Rep*. **7** (2017). 10.1038/s41598-017-01996-7 PMC5431860 28500297

[31] Christe P, Bernasconi M, Vontobel P, Turberg P, Parriaux A (2007). Three-dimensional petrographical investigations on borehole rock samples: A comparison between X-ray computed- and neutron tomography. Acta Geotech..

[32] Perfect E (2014). Neutron imaging of hydrogen-rich fluids in geomaterials and engineered porous media: A review. Earth-Science Reviews.

[33] Kardjilov N, Hilger A, Manke I, Woracek R, Banhart J (2016). CONRAD-2: The new neutron imaging instrument at the Helmholtz-Zentrum Berlin. J. Appl. Crystallogr..

[34] Permanyer A, Douifi L, Lahcini A, Lamontagne J, Kister J (2002). FTIR and SUVF spectroscopy applied to reservoir compartmentalization: A comparative study with gas chromatography fingerprints results. Fuel.

[35] Pernyeszi T, Patzkó Á, Berkesi O, Dékány I (1998). Asphaltene adsorption on clays and crude oil reservoir rocks. Colloids Surfaces A Physicochem. Eng. Asp..

[36] Henke BL, Gullikson EM, Davis JC (1993). X-ray interactions: Photoabsorption, scattering, transmission, and reflection at E = 50–30, 000 eV, Z = 1–92. At. Data Nucl. Data Tables.

[37] Kardjilov, N. Further developments and applications of radiography and tomography with thermal and cold neutrons. (2003).

[38] Kardjilov, N. *Further developments and applications of radiography and tomography with thermal and cold neutrons*. (Technische Universität München, 2003).

[39] Neutron activation and scattering calculator. Available at, https://www.ncnr.nist.gov/resources/activation/. (Accessed: 15th October 2018)

[40] Mendoza de la Cruz JL (2009). Study of monolayer to multilayer adsorption of asphaltenes on reservoir rock minerals. Colloids Surfaces A Physicochem. Eng. Asp..

[41] Fogden A (2012). Removal of crude oil from kaolinite by water flushing at varying salinity and pH. Colloids Surfaces A Physicochem. Eng. Asp..

[42] Lebedeva EV, Fogden A (2011). Micro-CT and wettability analysis of oil recovery from sand packs and the effect of waterflood salinity and kaolinite. Energy and Fuels.

[43] Kumar M, Fogden A (2010). Patterned wettability of oil and water in porous media. Langmuir.

[44] Wei B (2018). Relation between brine-crude oil-quartz contact angle formed on flat quartz slides and in capillaries with brine composition: Implications for low-salinity waterflooding. Colloids. Surfaces A Physicochem. Eng. Asp..

[45] Tötzke, C., Kardjilov, N., Manke, I. & Oswald, S. E. Capturing 3D Water Flow in Rooted Soil by Ultra-fast Neutron Tomography. *Sci*. *Rep*. **7** (2017). 10.1038/s41598-017-06046-w PMC5522441 28733616

[46] Cnudde V, Boone MN (2013). High-resolution X-ray computed tomography in geosciences: A review of the current technology and applications. Earth-Science Reviews.

[47] Görner W (2001). BAMline: The first hard X-ray beamline at BESSY II. in Nuclear Instruments and Methods in Physics. Research, Section A: Accelerators, Spectrometers, Detectors and Associated Equipment.

