# Supplementary material for: Probing the 3D molecular and mineralogical heterogeneity in oil reservoir rocks at the pore scale
Source: Sci Rep. 2019 Jun 4;9:8263. doi: 10.1038/s41598-019-44763-6 (PMC6547720; doi:10.1038/s41598-019-44763-6)
Supplement: Supplementary file 1 — Supplementary Information [file 41598_2019_44763_MOESM1_ESM.docx]

Supplementary Information for

**Probing the 3D molecular and mineralogical heterogeneity in oil reservoir rocks at the pore scale**

Guilherme José Ramos Oliveira, Paula Campos de Oliveira, Rodrigo Surmas, Leandro de Paulo Ferreira, Henning Markötter, Nikolay Kardjilov, Ingo Manke, Luciano Andrey Montoro, Augusta Isaac

**This PDF file includes:**

Figures. S1 and S2

Table S1 and S2


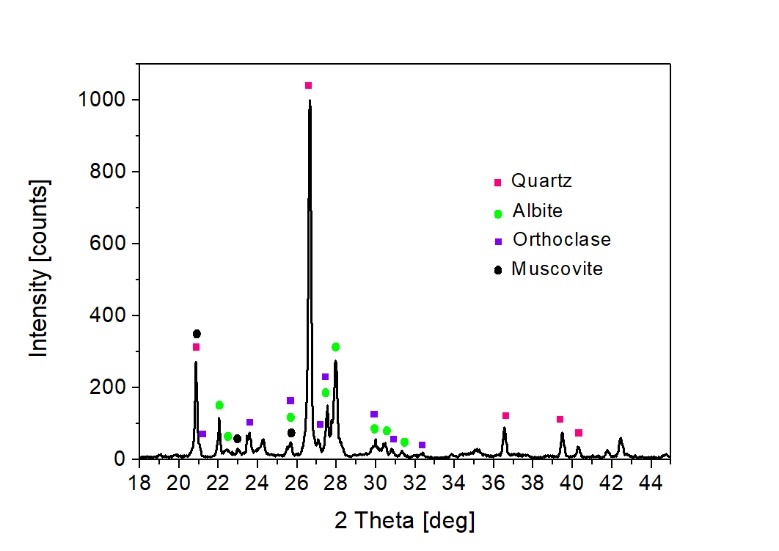


**Figure S1.** Diffraction pattern of the sandstone rock sample. The mineral phases present in the rock sample are quartz, albite, orthoclase, and muscovite.

**
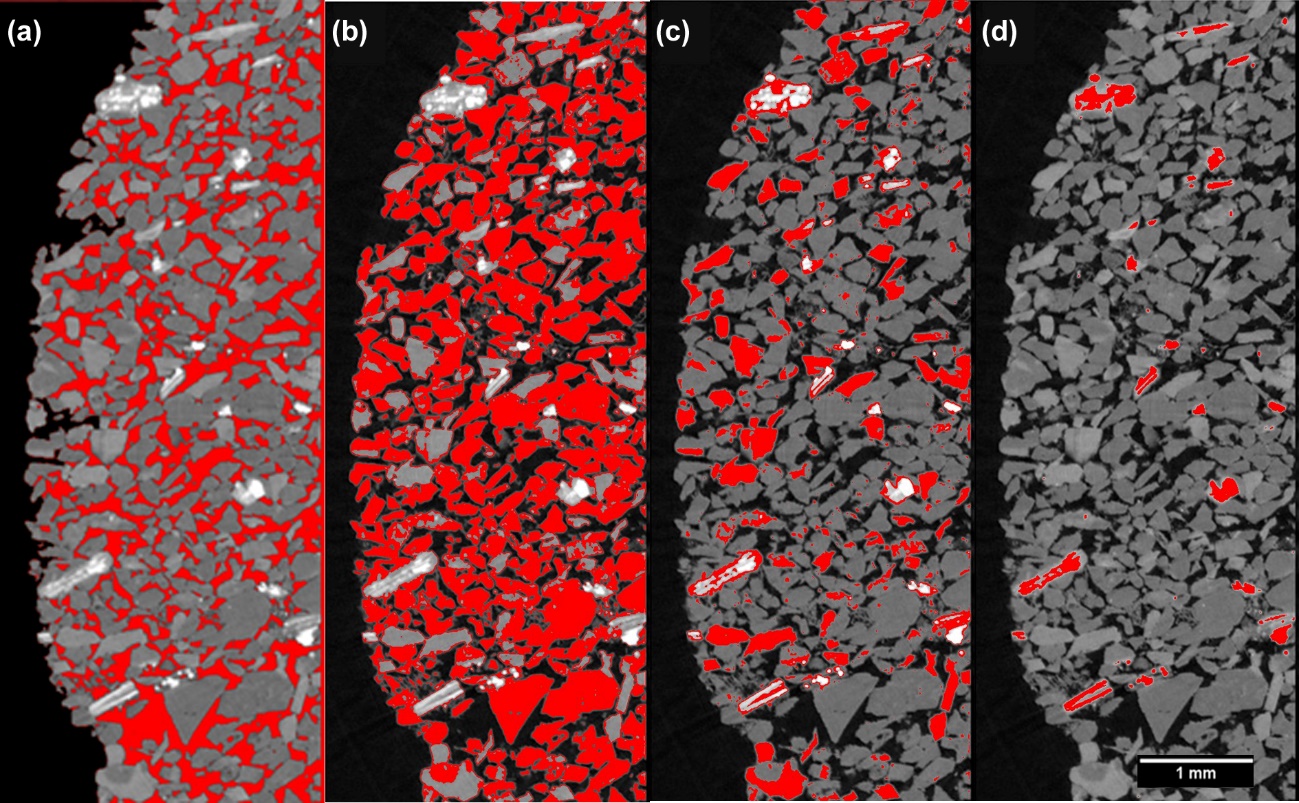
**

**Figure S2.** Segmentation process of the X-ray images. The segmented phases are highlighted in red: (a) rock porosity, (b) quartz and albite, (c) orthoclase, and (d) mica grains and heavy inclusions.

**Table S1.** Quantitative phase analysis of the sandstone rock based on the X-ray tomography and X-ray diffraction results.

| Segmented phases | | Volume fraction (%) | |
| --- | --- | --- | --- |
|  |  | **X-ray tomography** | **X-ray diffraction** |
| Minerals | **Quartz** | 68.2 | 39.9 |
|  | **Albite** |  | 30.3 |
|  | **Orthoclase** | 28.9 | 30.4 |
|  | **Muscovite** | 2.9 | - |

**Table S2.** Theoretical attenuation coefficients for neutrons (Σ_th_) of the main constituents of the sandstone rock sample. Data obtained from Physical Measurement Laboratory, National Institute of Standards and Technology (<http://physics.nist.gov>).

| **Mineral Phase** | **Formula** | **Density**  **[g/cm^3^]** | **Σ_th_**  **[cm^-1^]** |
| --- | --- | --- | --- |
| **Quartz** | SiO_2_ | 2.65 | 0.287 |
| **Albite** | NaAlSi_3_O_8_ | 2.62 | 0.280 |
| **Orthoclase** | KAlSi_3_O_8_ | 2.56 | 0.260 |
| **Muscovite** | KAl_3_Si_3_O_10_(OH)_2_ | 2.82 | 0.935 |
